# Effect of Ezetimibe on Glucose Metabolism and Inflammatory Markers in Adipose Tissue

**DOI:** 10.3390/biomedicines8110512

**Published:** 2020-11-18

**Authors:** Yongin Cho, Ryeong-Hyeon Kim, Hyunki Park, Hye Jin Wang, Hyangkyu Lee, Eun Seok Kang

**Affiliations:** 1 Department of Endocrinology and Metabolism, Inha University School of Medicine, Incheon 22332, Korea; choyorin@gmail.com; 2 Graduate School, Yonsei University College of Medicine, Seoul 03722, Korea; 3 Division of Endocrinology and Metabolism, Department of Internal Medicine, Yonsei University College of Medicine, Seoul 03722, Korea; rhkim@yuhs.ac (R.-H.K.); hyejin003300@naver.com (H.J.W.); 4 Biobehavioral Research Center, Mo-Im Kim Nursing Research Institute, Yonsei University College of Nursing, Seoul 03722, Korea; maniam@naver.com (H.P.); hkyulee@yuhs.ac (H.L.)

**Keywords:** ezetimibe, glucose metabolism, adipocyte, insulin resistance

## Abstract

Despite numerous studies, the effects of ezetimibe on glucose metabolism are poorly understood. Here, we aimed to investigate the effects of ezetimibe on glucose metabolism and the expression of inflammatory markers. Thirteen rats were randomly assigned to an ezetimibe (*n* = 6) or control group (*n* = 7). The control group received a high fat diet (HFD; 60 Kcal%), whereas the ezetimibe group received an HFD (60 Kcal%) containing 160 mg/kg of ezetimibe. After 14 weeks, adipose and liver tissues, along with plasma, were collected and comparatively analyzed. The effects of combination therapy with ezetimibe and statins on glucose metabolism were investigated over a 1-year period using data from patients with hyperlipidemia. Several indices of glucose metabolism partially improved in the ezetimibe group. The sizes of adipocytes and the accumulation of pro-inflammatory cytokines were reduced in the ezetimibe group. Ezetimibe treatment induced anti-inflammatory cytokines and fatty acid oxidation in adipocytes and reduced serum levels of free fatty acids. Clinical data analysis revealed that statin monotherapy significantly increased insulin resistance. However, combination therapy with ezetimibe and statins did not increase insulin resistance. In conclusion, ezetimibe was found to reduce the sizes of adipocytes in visceral fat and serum levels of free fatty acids, to induce fatty acid oxidation, to improve adipocytic inflammation, and to partially improve glycemic index values.

## 1. Introduction

Ezetimibe is a hyperlipidemic drug that selectively inhibits cholesterol absorption by binding to the cholesterol carrier, Niemann-Pick C1 like 1 (NPC1L1), which is present in intestinal membranes. Ezetimibe lowers the serum levels of cholesterol by suppressing the absorption of intestinal cholesterol. It can be used as a supplementary or alternative drug when the hypolipidemic effects of statins, 3-hydroxy-3-methylglutaryl coenzyme A reductase inhibitors, are insufficient or their usage is contraindicated. In a recently published large-scale clinical study (the IMPROVE-IT trial), ezetimibe significantly improved hypercholesterolemia and reduced cardiovascular disease in combination with statins; moreover, it reduced the relative risks of cardiovascular events by 6.4%, compared to statin monotherapy [1].

The use of statins has been linked to an increased risk of diabetes, and high-dose statin therapy has been shown to be associated with an increased risk of new-onset diabetes, compared to treatment with moderate doses of statins [2]. Several studies have indicated that statins increase serum glucose levels and insulin resistance by enhancing hepatic glucose production, disrupting intracellular insulin signaling, and hindering glucose uptake in peripheral tissues [3,4,5,6,7]. Meanwhile, replacing high-dose statin therapy with ezetimibe (alone or in combination with low-dose statins) has been found to reduce the risk of hyperglycemia and the other side effects of statins, including myotoxicity and hepatotoxicity [8]. However, the effects of ezetimibe on glucose metabolism are poorly understood. Although several studies have demonstrated that ezetimibe improves insulin resistance and reduces visceral fat [9,10], some studies have reported that the use of ezetimibe increases blood glucose levels [11]. Research has also demonstrated that hepatic steatosis, which is closely related to insulin resistance, is improved following ezetimibe treatment in some animal models [12], but not in human subjects [13].

There are several plausible explanations for these conflicting observations. The expression of hepatic NPC1L1 is abundant in humans, but is very low in mice [14], and its role in glucose metabolism remains unclear. This may have produced different results, as the effects of ezetimibe on glucose metabolism may differ between mice and humans. Additionally, considering the mechanism by which ezetimibe inhibits the absorption of dietary cholesterol, we suspect that effects may vary depending on the amount of dietary cholesterol or the degree of metabolic stress.

In this study, we aimed to investigate the effects of ezetimibe on glucose metabolism both in vitro and in vivo by administering a high fat diet (HFD) to rats expressing hepatic NPC1L1, similar to humans. By identifying the molecular mechanisms underlying the effects of ezetimibe, we aimed to determine whether ezetimibe therapy can be safely administered to patients with diabetes and hepatic steatosis.

## 2. Experimental Section

### 2.1. In Vivo Study

As a pilot study, 4-week-old C57BL/6J male mice (*n* = 39) were fed a standard diet (5% wt/wt fat) for 1 week to adapt to the environment. At 5 weeks of age, mice were randomly assigned to receive HFD with ezetimibe (45 Kcal% diet containing 0.004% w/w ezetimibe) in the ezetimibe group (*n* = 21) or a HFD (45 Kcal%) in the control group (*n* = 18). The total observation period was 19 weeks. Weight, dietary intake, activity patterns, and health status were monitored daily throughout the experiment. Fasting blood glucose levels were measured at baseline and after 19 weeks of drug administration. Oral glucose tolerance test was performed after 17 weeks of drug administration, when diabetes developed. Insulin tolerance test was performed 1 week after oral glucose tolerance test (OGTT) (after 18 weeks of drug administration). To investigate whether ezetimibe has a direct effect on mice liver, the expression of NPC1L1 was tested in both mice liver and HepG2 cells.

In the primary study, 5-week-old male Wistar rats (*n* = 13) were housed under standard conditions at a temperature of 21 ± 2 °C and relative humidity of 60 ± 10% under a 12 h light/dark cycle, with ad libitum access to food and water. The rats were randomly assigned to an ezetimibe (*n* = 6) or control group (*n* = 7) at 6 weeks of age. The control group received an HFD (60 Kcal%), whereas the ezetimibe group received an HFD (60 Kcal%) containing 160 mg/kg ezetimibe. The concentration of ezetimibe (estimated 6.4 mg/kg/day) was determined based on previous experiments in rats [12,15,16]. The total observation period was 14 weeks. Body weight, food intake, activity patterns, and health status of the animals were monitored on a daily basis throughout the duration of the experiment. All animal procedures were performed in accordance with the guidelines of the National Institutes of Health and pre-approved by the committee for animal care and use of Yonsei University, College of Medicine (2017-0028).

An OGTT was performed after 12 weeks of administering ezetimibe. After overnight fasting (18 h) [17], 2 g/kg of glucose was orally administered, and blood glucose levels were measured from caudal venous blood using a portable blood glucose meter (Boehringer-Mannheim, Indianapolis, IN, USA) after 0, 15, 30, 60, 90, and 120 min of glucose administration. An insulin tolerance test (ITT) was performed after 10 weeks of administering ezetimibe. After 4 h of fasting [18], 1 U/kg of insulin (Sigma-Aldrich, Cat. No. 9177C) was administered intraperitoneally, and blood glucose levels were measured from the caudal venous blood using a portable blood glucose meter after 0, 15, 30, 60, 90, and 120 min of administering insulin. Two weeks after the OGTT, anesthesia was performed after 18 h of fasting using a nose cone. Blood was obtained from the abdominal aorta via a thoracotomy, after which the rats were euthanized. The blood samples were centrifuged (4000 rpm) for 10 min, and the serum was stored at −80 °C. The liver and adipose tissues were excised after euthanasia. Serum fasting glucose, stimulated glucose, fasting insulin, stimulated insulin, aspartate aminotransferase (AST), alanine aminotransferase (ALT), total cholesterol, triglyceride, and free fatty acid levels were determined.

Tissue samples were washed, dehydrated, and embedded in paraffin. Some of the tissue sections were stained with hematoxylin and eosin for histological analyses. Tissue samples were examined under a microscope, and images were acquired using an attached digital camera. The diameters of 100 random adipocytes in each rat were measured. CellSens Entry software (Olympus, Tokyo, Japan) was used for image analysis.

### 2.2. In Vitro Study

The effect of ezetimibe on glucose metabolism in HepG2 cells was compared with that in cells in controls on the basis of the assumption that ezetimibe directly acts on hepatic NPC1L1. Ezetimibe was dissolved in dimethyl sulfoxide before adding it to the culture medium. In all experiments, ezetimibe was administered at a final concentration of 25 µM, and the final concentration of dimethyl sulfoxide was ≤0.1%. The details of the materials and methods are available in the online-only Supplementary Materials and Appendix A.

### 2.3. Human Study

Patient data were reviewed to investigate the effects of ezetimibe on insulin resistance in human subjects. We reviewed the electronic medical records of patients at Severance Hospital (a tertiary care university hospital in Seoul, Korea). The patients were aged ≥19 years, had dyslipidemia, and had initiated treatment for dyslipidemia. The fasting insulin and fasting glucose levels of the patients were tested before and after 1 year of pharmacotherapy between January 2006 and December 2018. Patients in whom their antidyslipidemic or anti-diabetic medications had been altered during the observation period were excluded from the study. Finally, data for 133 patients who satisfied the above conditions were collected.

Age, sex, weight, height, status of diabetes, and current medications were recorded. Body mass index values were calculated by dividing body weight by the square of the height (kg/m^2^). Following an overnight fast of ≥8 h, blood samples were collected before (0 min, designated as ‘fasting’) and after (120 min, designated as ‘stimulated’) meals to measure HbA1c, basal, glucose, stimulated glucose, fasting insulin, and other biochemical parameters. Insulin sensitivity was assessed using the homeostasis model assessment of insulin resistance (HOMA-IR) index [19]. The Ethics Committee of the Yonsei University College of Medicine approved this study (4-2018-1038).

### 2.4. Statistical Analysis

All categorical variables are expressed numerically as a proportion and were compared by χ^2^ analysis. Continuous variables were compared using the Wilcoxon-Mann-Whitney U test or Kruskal-Wallis test. The Wilcoxon signed-rank test was used to compare the clinical data before and after treatment. All statistical analyses were performed using IBM SPSS statistical software for Windows, version 25.0 (IBM, Armonk, NY, USA). For statistical analysis, *p* values <0.05 were considered statistically significant.

## 3. Results and Discussion

We performed a pilot study in mice, and details on the materials and methods thereof are available as an online-only supplement. Therein, treatment with ezetimibe did not significantly affect overall glycemic patterns. However, ITTs revealed a significant reduction in blood glucose levels in the ezetimibe group at 30 and 60 min, compared to those in the control group (Supplementary Figure S1). In order to investigate whether ezetimibe has direct effects on mice liver, the expression of NPC1L1 was determined in both mice liver tissue and HepG2 cells. As depicted in Supplementary Figure S2a, there was no evidence of the expression of NPC1L1 in the mice liver. From the results of this pilot study, we hypothesized that ezetimibe could improve insulin resistance without directly acting on the liver. Further, we confirmed the hepatic expression of NPC1L1 in HepG2 cells or rat liver (Supplementary Figure S2b). NPC1L1 is not only present in the intestinal membrane, but also in hepatocytes. It is involved in the reabsorption of cholesterol released into bile [20]. The role and mechanisms of action of hepatic NPC1L1 are yet to be fully understood. Although NPC1L1 is rarely expressed in mice hepatocytes, it is expressed in the liver of both rats and humans [14]. Thus, we investigated the effect of ezetimibe on glucose metabolism following the administration of HFD in a rat model with the hepatic expression of NPC1L1, similar to human subjects.

**Figure 1:**
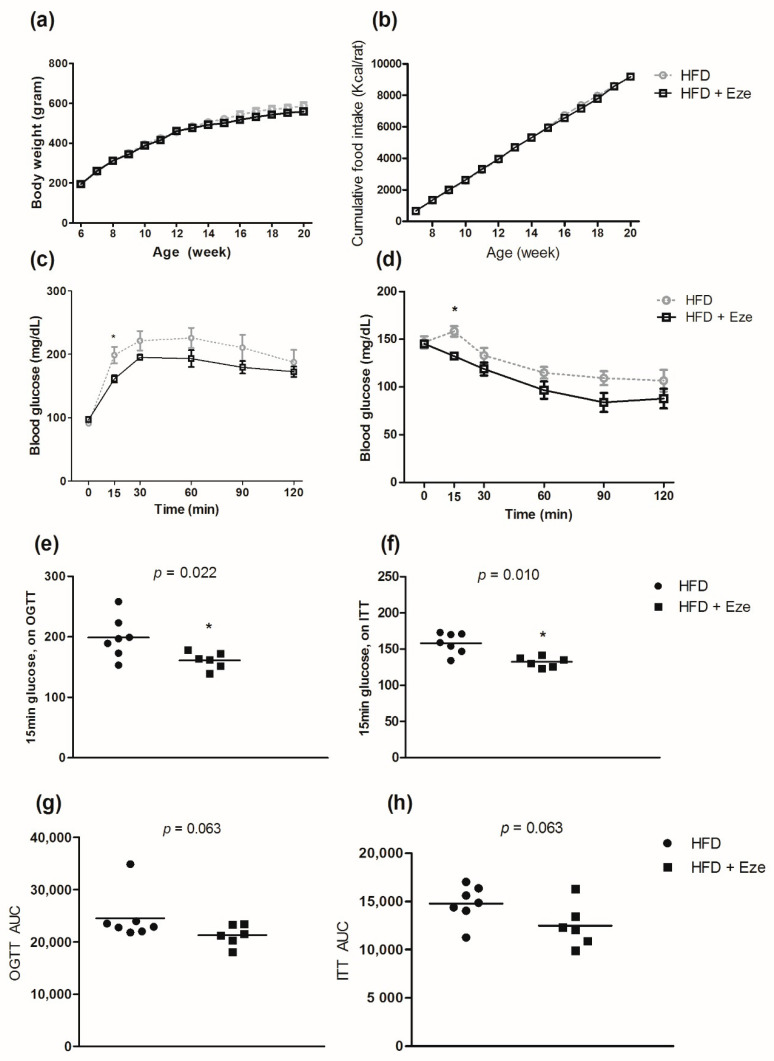
Effects of ezetimibe on oral glucose tolerance test (OGTT) and insulin tolerance test (ITT). (**a**) Gain in body weight, (**b**) cumulative food intake (Kcal/animal) (**c**) OGTT, (**d**) ITT, (**e**) OGTT after 15 min of glucose administration, (**f**) ITT after 15 min of glucose administration, (**g**) AUC of OGTT, and (**h**) AUC of ITT. Compared using the Wilcoxon-Mann-Whitney U test; * *p* < 0.05. OGTT; oral glucose tolerance test, ITT; insulin tolerance test.

The characteristics of the rat model are described in Supplementary Table S1. There was no difference in the body weights between the experimental groups at baseline. At the end of the treatment period (14 weeks), there was no difference in body weight or body weight gain between the groups. Although liver weight decreased significantly in the ezetimibe group (11.2 ± 1.0 g), compared to that in the control group (13.0 ± 1.4 g) (*p* = 0.015), there was no significant difference in the liver weight, total body weight (%), or weight of the perigonadal fat between the ezetimibe and control groups (all *p* > 0.05). Over the 14-week study period, there was no difference in body weight gain between the ezetimibe and control groups (Figure 1a), and there was no statistical difference in overall food intake between the groups (Figure 1b). In the rat model, the results of OGTTs revealed that glucose tolerance in the ezetimibe group significantly improved after 15 min of glucose administration, compared to that in the control group (*p* < 0.022, Figure 1c,e). The AUC value of the OGTT was lower for the ezetimibe group than for the control group; however, the difference was not statistically significant (Figure 1g, *p* = 0.063). Insulin tolerance in the ezetimibe group improved more after 15 min of insulin administration, compared to that in the control group (*p* = 0.010, Figure 1d,f). The AUC value of ITTs was lower for the ezetimibe group than for the control group; however, the difference was not statistically significant (Figure 1h, *p* = 0.063).

**Figure 2:**
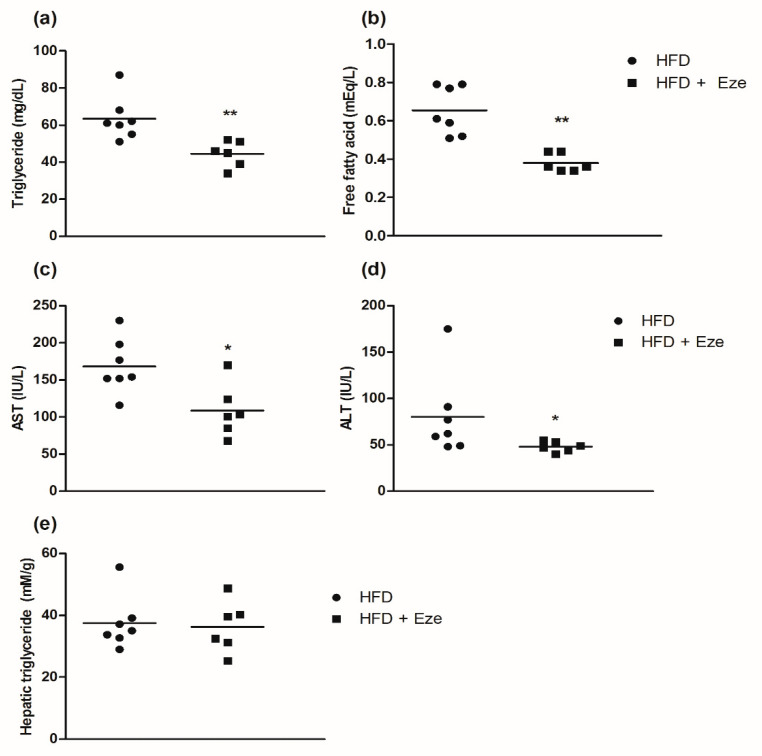
Effects of ezetimibe on triglyceride levels, free fatty acid levels, aspartate aminotransferase (AST) levels, alanine aminotransferase (ALT) levels, and hepatic triglyceride levels. (**a**) Triglyceride levels, (**b**) free fatty acid levels, (**c**) AST (IU/L) levels, (**d**) ALT (IU/L) levels, and (**e**) hepatic triglyceride (mM/g) levels. The hepatic triglyceride levels were measured using a triglyceride quantification kit (Abcam, Cat. No. ab65336). Compared using the Wilcoxon-Mann-Whitney U test; * *p* < 0.05, ** *p* < 0.01. AST; aspartate aminotransferase, IU/L; international units per liter, ALT; alanine aminotransferase.

Serum levels of triglycerides and free fatty acids were significantly lower in the ezetimibe group than in the control group (all *p* < 0.01, Figure 2a,b). The levels of aspartate aminotransferase and alanine aminotransferase decreased in the ezetimibe group (all *p* < 0.05, Figure 2c,d). However, the difference in the levels of hepatic triglycerides between the ezetimibe and control groups was not statistically significant (*p* = 0.886, Figure 2e).

Several of the glucose metabolism indices partially improved in the rats in the ezetimibe group. However, these differences did not lower systemic blood glucose levels, and the observed improvement in hepatic steatosis was not statistically significant. The features of non-alcoholic fatty liver disease are closely related to those of metabolic syndrome, including obesity and insulin resistance. Improvement in insulin resistance inhibits sterol regulatory element binding protein-1c and fatty acid synthase [21], which improve hepatic steatosis. A few studies have reported that fatty liver also improves in human subjects following ezetimibe treatment [11,22]. However, the findings of some studies on improvement in fatty liver in human subjects following ezetimibe therapy are unclear. By measuring hepatic fat fractions by magnetic resonance imaging proton density, Loomba and colleagues reported no significant improvement in hepatic steatosis following ezetimibe treatment [13]. In the present study, the experimental period of the present study (14 weeks) may not have been long enough to induce hepatic steatosis; therefore, further studies conducted over a longer study period are necessary.

**Figure 3:**
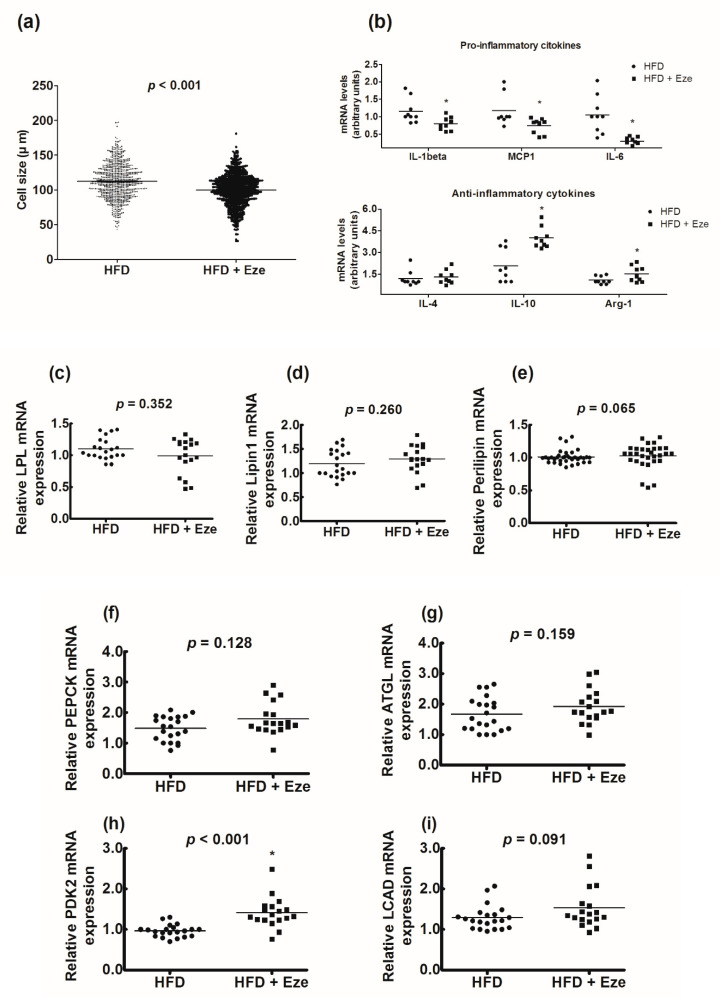
Effects of ezetimibe on the sizes of adipocytes, markers of inflammation, and genes involved in lipogenesis, lipolysis, and β-oxidation. (**a**) The effects of ezetimibe on the sizes of adipocytes were compared using two-sample t-test for a total of 1,300 adipocytes (*n* = 100 per animal). The sizes of the adipocytes were markedly reduced in the ezetimibe group, compared to those in the control group (*p* < 0.001). (**b**) The effects of ezetimibe on markers of inflammation: (upper panel) pro-inflammatory cytokines and (lower panel) anti-inflammatory cytokines. (**c**) LPL, (**d**) Lipin1, (**e**) Perilipin, (**f**) PEPCK, (**g**) ATGL, (**h**) PDK2, and (**i**) LCAD. Compared using the Wilcoxon-Mann-Whitney U test; * *p* < 0.05. LPL; lipoprotein lipase, PEPCK; phosphoenolpyruvate carboxykinase, ATGL; adipose triglyceride lipase, PDK2; pyruvate dehydrogenase kinase-2, LCAD; long-chain acyl-CoA dehydrogenase.

The sizes of adipocytes in the ezetimibe group (99.8 ± 23.4 µm) were significantly smaller than those in the control group (112.2 ± 25.1 µm) (*p* < 0.001, Figure 3a). The levels of pro-inflammatory cytokines IL-1β, MCP1, and IL-6 significantly decreased in the ezetimibe group (all *p* <0.05). Conversely, the levels of anti-inflammatory cytokines IL-10 and Arg-1 significantly increased in the ezetimibe group (all *p* < 0.05, Figure 3b). There was no significant change in the mRNA levels of the genes associated with lipogenesis (Figure 3c–e) or lipolysis (Figure 3f–g) following ezetimibe treatment. However, the mRNA levels of pyruvate dehydrogenase kinase-2, which is associated with fatty acid oxidation, significantly increased following treatment with ezetimibe (*p* < 0.001, Figure 3h). The expression of long-chain acyl-CoA dehydrogenase, which plays an important role in β-oxidation, also tended to increase, however, the change was not statistically significant (Figure 3i).

Cholesterol, which is absorbed by the intestines, is transferred as chylomicrons to the blood via lymphatic circulation and is distributed throughout the body via systemic circulation. Chylomicron levels increase after meals and contribute to the development of atherosclerosis. This reduces the expression of low-density lipoprotein (LDL) receptors in the liver and increases the levels of LDL cholesterol [15]. Lipid overload in obesity is associated with adipocyte dysfunction, inflammation, macrophage infiltration, and decreased fatty acid oxidation [23]. A previous study on monkeys demonstrated that the sizes and inflammatory status of visceral adipocytes change when the dietary intake of cholesterol is increased [24], and as expected, the action of ezetimibe, limiting the intestinal absorption of dietary cholesterol, enhanced fatty acid oxidation and relieved inflammatory reactions in visceral adipose tissues in this study. Previous studies have reported that the accumulation of cholesterol in adipocytes is correlated with an increased risk of metabolic syndrome and cardiovascular diseases [25] and that the use of ezetimibe reduces cardiovascular risks [1]. While the cardiovascular benefits of ezetimibe are primarily mediated by lowering LDL cholesterol levels [1], the results of the current study suggest that a reduction in cholesterol accumulation in adipocytes may also reduce cardiovascular risk.

In this study, we observed that insulin resistance improved and that the levels of free fatty acids decreased following ezetimibe treatment. Free fatty acids induce insulin resistance in peripheral tissues [26]. Previous studies have demonstrated that ezetimibe inhibits not only dietary cholesterol absorption, but also dietary free fatty acids absorption [27]. Additionally, an increase in adipocytic inflammation has been shown to be associated with adipocyte hypertrophy, which is associated with increased insulin resistance [28]. These actions may be particularly beneficial for patients with diabetes. A meta-analysis by Hong and coworkers reported that ezetimibe-statin combination therapy is associated with greater cardiovascular benefits in patients with diabetes than in those without diabetes [29].

To investigate whether ezetimibe has direct effects on the liver, we evaluated phosphorylation levels of Akt in HepG2 cells after 15 min of stimulation with insulin to analyze the direct effects of ezetimibe on the hepatic insulin response. The difference in the levels of Akt phosphorylation in the HepG2 cells in the control and ezetimibe groups was not statistically significant (all *p* > 0.05, Supplementary Figure S3a). We evaluated the levels of glucose 6-phosphatase and phosphoenolpyruvate carboxykinase following stimulation with low (5.5 mM) concentrations of glucose to analyze the direct effect of ezetimibe on gluconeogenesis. Western blotting revealed that there was no significant difference in the levels of G6pase or PEPCK in the HepG2 cells in the control and ezetimibe groups (all *p* > 0.05, Supplementary Figure S4). However, PCR analyses revealed that the levels of G6pase and PEPCK significantly increased at several instances during ezetimibe treatment (Supplementary Figure S3b,c). PCR analyses revealed that hepatic glucose outflow increased significantly following ezetimibe treatment (Supplementary Figure S5).

In this study, the results of in vitro experiments revealed that insulin signaling and gluconeogenesis in hepatocytes did not improve significantly following ezetimibe treatment; however, gluconeogenesis and glucose outflow in human hepatocytes increased in the ezetimibe treatment group. This demonstrated that an increase in gluconeogenesis resulting from the direct action of ezetimibe on the liver may ameliorate its beneficial effects on systemic glucose metabolism. Although we observed an improvement in insulin resistance, the overall effect of ezetimibe on dysglycemia was not significant. In previous mouse model studies, the use of ezetimibe was associated with an improvement in dysglycemia [30]. However, the effects of ezetimibe on glucose metabolism has not been clearly reported in human subjects. Some randomized controlled trials on ezetimibe had to be discontinued owing to increases in blood glucose levels during the study period [11]. Kurano and coworkers reported that hepatic NPC1L1 is involved in suppressing gluconeogenesis [31]. In humans rich in hepatic NPC1L1 expression, the use of ezetimibe may lead to an increase in hepatic gluconeogenesis. This could be the reason why the use of ezetimibe appears to improve insulin resistance, but does not elicit a significant improvement in dysglycemia

We further investigated whether the observed effect of ezetimibe on glycemic indices can be confirmed through clinical data. In total, 133 patients with dyslipidemia who had initiated medication for dyslipidemia and had undergone tests for both fasting insulin and fasting glucose levels before and after 1 year of pharmacotherapy were included in this study. The patients were classified into the statin monotherapy group (*n* = 90, 67.7%) and the ezetimibe combination group (*n* = 43, 32.3%). The latter was sub-classified into ezetimibe add-on statin (*n* = 13, 9.8% of total) and ezetimibe start with statin (*n* = 30, 22.6% of total) subgroups. The baseline characteristics of the patients are summarized in Table 1. The study population included 66.9% women, and 11.3% of the patients had type 2 diabetes. All of the patients were of Asian descent. Both groups had similar baseline characteristics, with the exception of fasting glucose, total cholesterol, and levels of LDL cholesterol. There were differences in the cholesterol levels at the beginning of each treatment, possibly owing to the fact that each of the drugs had different indications: fasting glucose levels were higher in the ezetimibe add-on statin group than in the statin monotherapy group (all *p* < 0.05, Table 1).

There was a significant reduction in total cholesterol and LDL cholesterol levels in both groups, compared to those at baseline (all *p* < 0.05, Supplementary Table S2). The levels of fasting glucose, stimulated glucose, fasting insulin, and HOMA-IR significantly increased in the statin monotherapy group (all *p* < 0.05, Table 2). Intention-to-treat analysis revealed that there were no differences between the groups with respect to all metabolic indices (all *p* > 0.05). Insulin resistance significantly increased in the statin monotherapy group after 1 year (+0.33, *p* = 0.002); however, there was no significant increase in insulin resistance in the ezetimibe combination group (+0.14, *p* = 0.530). Additionally, insulin resistance in the ezetimibe add-on statin group tended to decrease, as calculated by HOMA-IR (−0.24, *p* = 0.382, Supplementary Figure S6).

The analysis of clinical data obtained from human subjects revealed that statin monotherapy significantly increased insulin resistance. In contrast, the use of ezetimibe in combination with statins did not increase insulin resistance. A recent meta-analysis revealed that ezetimibe treatment does not improve dysregulated glycemic control in human subjects [32]. However, it also has been reported that the use of ezetimibe improves glucose-related indicators, including insulin resistance [33] and visceral fat [9]. In addition, through a combination with ezetimibe, a sufficient cholesterol lowering effect can be expected even when a relatively small dose of statin is used [34]. Therefore, improved glucose metabolism through statin reduction can also be expected with the inclusion of ezetimibe.

This study has several limitations. First, insulin secretion function by studying pancreatic beta cells or the effects of ezetimibe on skeletal muscle, which consumes a high portion of glucose in the body, were not evaluated in this study. Since the concentration of ezetimibe in rats was higher than that for human daily use (10 mg/day), the effect of ezetimibe on glucose metabolism in rats could be amplified. Second, the study population was not large enough; therefore, several analyses could not be performed. For this reason, we are unable to exclude the possibility that the statistically insignificant results obtained here may have clinical significance. Considering the relatively short duration of this study, further studies are necessary to determine whether the long-term use of ezetimibe improves dysglycemia. Third, owing to the limited expression of NPC1L1 in specific organs, it was difficult to explain how ezetimibe indirectly affected adipocytes. Additionally, the study on human subjects was retrospectively designed. Thus, important data, including body weight and waist circumference, were not determined to ensure that these data are not reflected in the results. However, the importance of this study lies in the fact that it is the first to describe changes in adipocytes following ezetimibe treatment, owing to its effect on glucose metabolism. The results of this study suggest that ezetimibe can be safely administered to patients with diabetes.

## 4. Conclusions

The use of ezetimibe reduced the sizes of adipocytes in visceral fat. Furthermore, ezetimibe reduced the accumulation of pro-inflammatory cytokines and induced the production of anti-inflammatory cytokines within adipocytes, leading to increased fatty acid oxidation; reduced levels of free fatty acids; and improved insulin resistance. However, there was no significant effect on systemic glucose control, which was confirmed from the retrospectively designed clinical study.

## Figures and Tables

**Table 1:** Baseline characteristics of the study population.

	Combination Therapy		Monotherapy	*p* Value
	Ezetimibe Add on Statin (*n* = 13)	Ezetimibe Start with Statin (*n* = 30)	Statin Monotherapy (*n* = 90)	
Age (years)	61.0 (16.5)	58.5 (11.8)	58.0 (10.0)	0.467
Female (%)	9 (69.2)	17 (56.7)	63 (70.0)	0.398
Diabetes (%)	2 (15.4)	3 (10.0)	10 (11.1)	0.873
BMI (kg/m^2^)	24.0 (6.9)	24.7 (3.9)	24.0 (3.7)	0.658
Glucose, fasting (mg/dL)	109.0 (27.0)	105.0 (15.3)	99.0 (17.0) *	0.008
Glucose, stimulated (mg/dL)	123.5 (79.0)	118.0 (40.0)	111.0 (30.0)	0.492
Insulin, fasting (uU/mL)	7.3 (9.2)	6.1 (3.1)	6.2 (3.6)	0.126
HOMA-IR	2.0 (2.5)	1.6 (0.9)	1.5 (0.9)	0.060
HbA1c (%)	6.1 (1.1)	5.8 (1.0)	5.9 (0.4)	0.110
AST (IU/L)	22.0 (5.5)	19.5 (6.3)	21.0 (8.0)	0.401
ALT (IU/L)	18.0 (17.5)	18.5 (7.8)	18.0 (9.5)	0.743
Total cholesterol (mg/dL)	184.0 (38.5)	259.0 (40.5) *	232.0 (47.5) *^,†^	<0.001
Triglycerides (mg/dL)	113.0 (73.5)	125.0 (96.5)	132.0 (68.0)	0.989
HDL cholesterol (mg/dL)	50.0 (14.0)	54.0 (13.5)	52.0 (15.0)	0.429
LDL cholesterol (mg/dL)	112.6 (29.6)	169.6 (36.2) *	150.2 (52.0) *^,†^	<0.001

BMI, body mass index; HOMA, homeostatic model assessment; IR, insulin resistance; AST, aspartate aminotransferase; ALT, alanine aminotransferase; HDL, high-density lipoprotein; LDL, low-density lipoprotein. Kruskal-Wallis test, Dunn procedure; NOTE. Bold text indicates *p* values < 0.05; * *p* values < 0.05 vs. ezetimibe add on statin group, by post hoc analyses (Dunn procedure); ^†^ *p* values < 0.05 vs. ezetimibe start with statin group, by post hoc analyses (Dunn procedure), *p* values < 0.05.

**Table 2:** Comparison of changes in the parameters of patients treated with ezetimibe combination or statins after 1 year.

	Ezetimibe Combination (n = 43)			Statin Monotherapy (n = 90)			Difference between Groups
	Baseline	Post-Treatment	*p* Value	Baseline	Post-Treatment	*p* Value	*p* Value
Glucose, fasting (mg/dL)	106.0 (18.0)	105.0 (20.0)	0.296	99.0 (17.0)	101.0 (17.3)	0.021	0.996
Glucose, stimulated (mg/dL)	118.0 (58.0)	127.0 (52.5)	0.946	111.0 (30.0)	132.0 (54.0)	0.016	0.117
Insulin, fasting (uU/mL)	7.0 (3.8)	7.3 (4.6)	0.828	6.2 (3.6)	7.3 (4.1)	0.003	0.066
HOMA-IR	1.7 (0.9)	1.8 (1.3)	0.923	1.5 (0.9)	1.8 (1.1)	0.002	0.109
HbA1c (%)	6.0 (0.9)	5.9 (0.8)	0.793	5.9 (0.4)	5.9 (0.3)	0.758	0.865
AST (IU/L)	21.0 (6.0)	23.0 (9.0)	0.003	21.0 (8.0)	23.0 (7.0)	0.009	0.825
ALT (IU/L)	18.0 (9.0)	23.0 (21.0)	0.012	18.0 (9.5)	21.0 (13.0)	0.003	0.694
Total cholesterol (mg/dL)	241.0 (61.0)	167.0 (47.0)	<0.001	232.0 (47.5)	168.5 (40.3)	<0.001	0.850
Triglycerides (mg/dL)	125.0 (84.0)	116.5 (71.0)	0.347	132.0 (68.0)	110.5 (76.8)	0.014	0.629
HDL cholesterol (mg/dL)	53.0 (15.0)	51.5 (18.3)	0.175	52.0 (15.0)	52.0 (16.3)	0.077	0.830
LDL cholesterol (mg/dL)	154.0 (60.4)	82.9 (38.8)	<0.001	150.2 (52.0)	90.0 (30.2)	<0.001	0.566

The data are presented as a median (IQR), and the *p* values were obtained from the Wilcoxon signed-rank test. The difference in *p* values was determined via intention-to-treat analysis.

## Supplementary Materials

The following are available online at https://www.mdpi.com/2227-9059/8/11/512/s1: Figure S1. Systemic glucose metabolism evaluated with oral glucose tolerance test and insulin tolerance test. Figure S2. The expression of hepatic NPC1L1 on mice and rat liver. Figure S3. Effects of ezetimibe on markers of gluconeogenesis in HepG2 cells, evaluated by Western blot. Figure S4. Effects of ezetimibe on glucose outflow in HepG2 cells. Figure S5. Changes in homeostatic model assessment of insulin resistance values. Figure S6. Changes of homeostatic model assessment of insulin resistance in patients of each group. Table S1. Characteristics of the animals in the control group (HFD) and ezetimibe group (HFD + ezetimibe). Table S2. Changes in glycemic indicators and cholesterol levels before and after treatment.

## Appendix A. Materials and Methods (Detailed)

### Appendix A.1. In Vivo Study

Thirteen 5-week-old male Wistar rats were housed under standard conditions (21 ± 2 °C, 60 ± 10% humidity, 12 h light/dark cycle) with *ad libitum* access to food and water. The rats were randomly assigned to either the ezetimibe group (*n* = 6) or control group (*n* = 7) at 6 weeks of age. The control group was fed a high fat diet (HFD; 60 Kcal%) and the ezetimibe group was fed a HFD (60 Kcal%) containing ezetimibe (160 mg/kg). The total observation period was 14 weeks. Weight, dietary intake, activity patterns, and health status were monitored daily throughout the experiment.

All animal procedures were performed in accordance with the guidelines of the National Institutes of Health and pre-approved by the animal care and use committee of Yonsei University, College of Medicine (2017-0028).

#### Appendix A.1.1. Oral Glucose Tolerance Test (OGTT) and Insulin Tolerance Test (ITT)

Oral glucose tolerance test (OGTT) was performed after 12 weeks of drug administration. After fasting for 18 h, 2 g/kg of glucose was administered orally, and blood glucose levels were measured from caudal venous blood using a portable blood glucose meter (Boehringer-Mannheim, Indianapolis, IN, USA) at 0, 15, 30, 60, 90, and 120 min post glucose administration.

Insulin tolerance test (ITT) was performed after 10 weeks of drug administration. After fasting for 4 h, 1 U/kg of insulin (Sigma-Aldrich, Cat. No. 9177C) was administered intraperitoneally, and blood glucose levels from caudal venous blood were measured by a portable blood glucose meter at 0, 15, 30, 60, 90, and 120 min post insulin administration.

#### Appendix A.1.2. Blood and Tissue Sampling

Two weeks after OGTT, after 18 h of fasting, anesthesia was performed using a nose cone. Blood was obtained from the abdominal aorta by thoracotomy. The rat was then euthanized. The blood was centrifuged for 10 min, and the serum was stored at −80 °C. Post euthanasia, the liver and fat tissue were excised. The liver and fat tissue samples were rapidly frozen with nitrogen solution and stored at −80 °C. Some liver and fat tissue samples were fixed in 4% paraformaldehyde (Tech & Innovation Co., Ltd., Chuncheon, South Korea, Cat. No. BPP-9004-004LR) solution for more than 48 h and used for histological analysis.

#### Appendix A.1.3. Measurement of Metabolic Parameters and Inflammation Markers (Blood)

Serum fasting glucose concentration, stimulated glucose, fasting insulin, stimulated insulin (Morinaga Ultra Sensitive Rat Insulin ELISA Kit [Morinaga, Yokohama, Japan, Cat. No. M1103]), aspartate aminotransferase (AST), alanine aminotransferase (ALT), total cholesterol, triglyceride, and free fatty acid levels were determined.

#### Appendix A.1.4. Hematoxylin and Eosin (H&E) Staining

Tissue samples were washed, dehydrated, and embedded in paraffin. Some were stained with hematoxylin and eosin (H&E) for observation of histological structures. Tissue samples were examined under a microscope, and images were acquired using an attached digital camera. CellSens Entry software (Olympus, Tokyo, Japan) was used for image analysis.

### Appendix A.2. In Vitro Study

The effect of ezetimibe on glucose metabolism in HepG2 cells was compared assuming a direct action of ezetimibe on hepatic NPC1L1. Hepatocellular carcinoma (HepG2) cell lines were cultured in Dulbecco’s Modified Eagle’s Medium (Thermo Scientific, Waltham, MA, USA, SH30243.01) containing 10% fetal bovine serum (Thermo Scientific, SH30071.03), 100 U/mL penicillin, and 100 mg/mL streptomycin (Thermo scientific, SV30010) in a 5% CO_2_ incubator at 37 °C.

Ezetimibe was dissolved in dimethyl sulfoxide before dilution in culture medium. In all experiments, the final ezetimibe concentration was 25 uM, and the final dimethyl sulfoxide concentration was ≤0.1%.

#### Western Blot

Cells and tissues were lysed in radioimmunoprecipitation assay buffer, containing of 20 mM tris (hydroxymethyl) aminomethane hydrochloride (pH 7.5), 150 mM sodium chloride (NaCl), 1 mM disodium ethylenediaminetetraacetate dihydrate, 1 mM ethylene glycol bis(-aminoethyl ether)-N,N,N′,N′-tetraacetic acid, 1% Nonidet P-40, 1% sodium deoxycholate, 2.5 mM sodium pyrophosphate, 1 mM beta-glycerophosphate, 1 mM sodium orthovanadate, 1 μg/mL leupeptin (BRI-9010-010M, Tech & Innovation Co., Ltd.), and protease inhibitor and phosphatase cocktail (Thermo Scientific, 78440). Protein samples were separated using 10% polyacrylamide gels and transferred to polyvinylidene fluoride membranes (Millipore, PVH00010). The membranes were incubated with primary antibodies to measure the expression of the following proteins: G6Pase (Santacruz, Cat. No. sc-25840), phosphoenolpyruvate carboxykinase (PEPCK, Santa Cruz Biotechnology, Cat. No. sc-32879), NPC1L1 (Invitrogen, Cat. No. PA1-16800), total Akt (Cell Signaling, Cat. No. 4691S), and p-Akt (Ser473, Cell Signaling, Cat. No. 4060S). Anti-mouse IgG, horseradish peroxidase (HRP)-linked antibody (Santa Cruz Biotechnology, Cat. No. sc-516102), and anti-rabbit IgG, HRP-linked antibody (Cell Signaling, 7074S) were used as secondary antibodies. NPC1L1 expression in liver tissue was also confirmed.

### Appendix A.3. Quantitative Real-Time Polymerase Chain Reaction

Total RNA was isolated from tissues and cells with TRIzol reagent (15596–018, Invitrogen, Carlsbad, CA, USA) following the manufacturer’s instructions, and 2 mg of total RNA was reverse transcribed to cDNA using the High Capacity cDNA Reverse Transcription kit (4368814, Applied Biosystems, Foster City, CA, USA). The resultant cDNA was amplified in the ABI 7500 sequence detection system (4350584, Applied Biosystems, Foster City, CA, USA) using Power SYBR Green PCR Master Mix (4367659, Applied Biosystems, Foster City, CA, USA) with the following cycling conditions: 40 cycles of 95 °C for 5 s, 58 °C for 10 s, and 72 °C for 20 s. Target gene expression was normalized to that of GAPDH (glyceraldehyde-3-phosphate dehydrogenase, and quantitative analyses were conducted using the △△cycle threshold method and StepOne Software version 2.2.2.

The primer sets used for quantitative polymerase chain reaction (qPCR) are as follows:

Rat Gapdh forward, 5′–ATGGCACAGTCAAGGCTGAGA–3′

Rat Gapdh reverse, 5′–CGCTCCTGGAAGATGGTGAT–3′

Rat IL-1 beta forward, 5′–CACCTCTCAAGCAGAGCACAG–3′

Rat IL-1 beta reverse, 5′–GGGTTCCATGGTGAAGTCAAC–3′

Rat MCP-1 forward, 5′–TGTCTCAGCCAGATGCAGTTAAT–3′

Rat MCP-1 reverse, 5′–CATCTTGCCAGTGAATGAGTAGC–3′

Rat IL-6 forward, 5′–GGGACTGA TGTTGTTGACAGCC–3′

Rat IL-6 reverse, 5′–CATATGTAA TTAAGCCTCCGACTTGTG–3′

Rat IL-4 forward, 5′–TCTCACGTCACTGACTGTA–3′

Rat IL-4 reverse, 5′–CTTTCAGTGTTGTGAGCGT–3′

Rat IL-10 forward, 5′–GTCATCGATTTCTCCCCTGTGA–3′

Rat IL-10 reverse, 5′–TTCATGGCCTTGTAGACACCTTT–3′

Rat Agr-1 forward, 5′–CCTATGCGTCATTTGGGTGG–3′

Rat Agr-1 reverse, 5′–TACACGATGTCCTTGGCAGA–3′

(HepG2 cell, Human)

Human PEPCK forward, 5′–GAGAGAACTCCAGGGTGCTG–3′

Human PEPCK reverse, 5′–TTGCTTCAAGGCAAGGATCT–3′

Human G6pase forward, 5′–TTTGGGATCCAGTCAACACA–3′

Human G6pase reverse, 5′–CAGATGGGGAAGAGGACGTA–3′

Human Gapdh forward, 5′–TTTGGGATCCAGTCAACACA–3′

Human Gapdh reverse, 5′–CAGATGGGGAAGAGGACGTA–3′
