# Supplementary material for: Effect of Ezetimibe on Glucose Metabolism and Inflammatory Markers in Adipose Tissue
Source: Biomedicines. 2020 Nov 18;8(11):512. doi: 10.3390/biomedicines8110512 (PMC7698625; doi:10.3390/biomedicines8110512)

Supplementary table 1. Characteristics of the animals in the control group (HFD) and ezetimibe group (HFD + ezetimibe)

The data are presented as the mean ± SD and compared using the Mann-Whitney U test.
Dunnett's post-hoc test. *p < 0.05 versus HFD group. HFD; high fat diet.

	HFD (n=7)	HFD + Ezetimibe (n=6)	*p value*
Baseline (6 weeks)			
Body weight (g)	195.6 ± 7.9	195.3 ± 8.2	0.830
At the end of treatment (20 weeks)			
Body weight (g)	588.0 ± 46.7	559.0 ± 33.1	0.174
Weight gain (g)	392.1 ± 43.0	363.5 ± 29.0	0.153
Liver weight (g)	13.0 ± 1.4	11.2 ± 1.0	0.015
Liver weight / Body weight (%)	3.3 ± 0.5	3.0 ± 0.0	0.171
Perigonadal Fat weight (g)	14.0 ± 1.7	12.8 ± 2.9	0.388
Perigonadal Fat weight / Body weight (%)	3.6 ± 0.5	3.7 ± 0.8	0.937

Data are expressed as mean ± SD and compared by Mann-Whitney U test
Dunnett's post-test. **p* < 0.05 versus HFD group. HFD; high fat diet

Supplementary table 2. Changes in glycemic indicators and cholesterol levels before and after treatment

Before	Glucose, fasting (mg/dl)	Insulin, fasting (uU/ml)	HOMA-IR	Total cholesterol (mg/dl)	Triglycerides (mg/dl)	LDL cholesterol (mg/dl)
Ezetimibe combination (n=43)	106.0 (97.0-115.0)	7.0 (4.8-8.6)	1.7 (1.3-2.2)	241.0 (205.0-266.0)	125.0 (93.0-177.0)	154.0 (119.4-179.8)
-ezetimibe add on statin (n=13)	109.0 (101.0-128.0)	7.3 (5.6-14.8)	2.0 (1.5-4.0)	184.0 (168.5-207.0)	113.0 (98.0-171.5)	112.6 (93.8-123.4)
-ezetimibe start with statin (n=30)	105.0 (96.5-111.8)	6.1 (4.8-7.9)	1.6 (1.3-2.1)	259.0 (233.0-273.5)	125.0 (90.0-186.5)	169.6 (150.8-186.9)
Statin monotherapy (n=90)	99.0 (91.0-108.0)	6.2 (4.4-8.0)	1.5 (1.1-2.0)	232.0 (203.0-250.5)	132.0 (99.0-167.0)	150.2 (116.2-168.2)
**After 1 year**						
Ezetimibe combination (n=43)	105.0 (97.0-117.0)	7.3 (4.7-9.3)	1.8 (1.1-2.4)	167.0 (152.0-199.0)	116.5 (88.8-159.8)	82.9 (72.4-111.2)
-ezetimibe add on statin (n=13)	105.0 (98.0-120.0)	8.0 (4.8-13.9)	1.9 (1.3-4.3)	167.0 (147.5-194.0)	112.0 (84.0-141.5)	90.8 (72.8-113.9)
-ezetimibe start with statin (n=30)	106.5 (96.8-114.3)	7.0 (4.3-8.9)	1.7 (1.1-2.4)	167.5 (151.3-207.3)	119.0 (89.0-168.0)	82.2 (72.0-104.4)
Statin monotherapy (n=90)	101.0 (93.0-110.3)	7.3 (5.1-9.2)	1.8 (1.3-2.4)	168.5 (150.0-190.3)	110.5 (82.3-159.0)	90.0 (76.4-106.6)

Data are expressed as median (IQR).

Supplementary figure 1. Systemic glucose metabolism evaluated with oral glucose tolerance test and insulin tolerance test

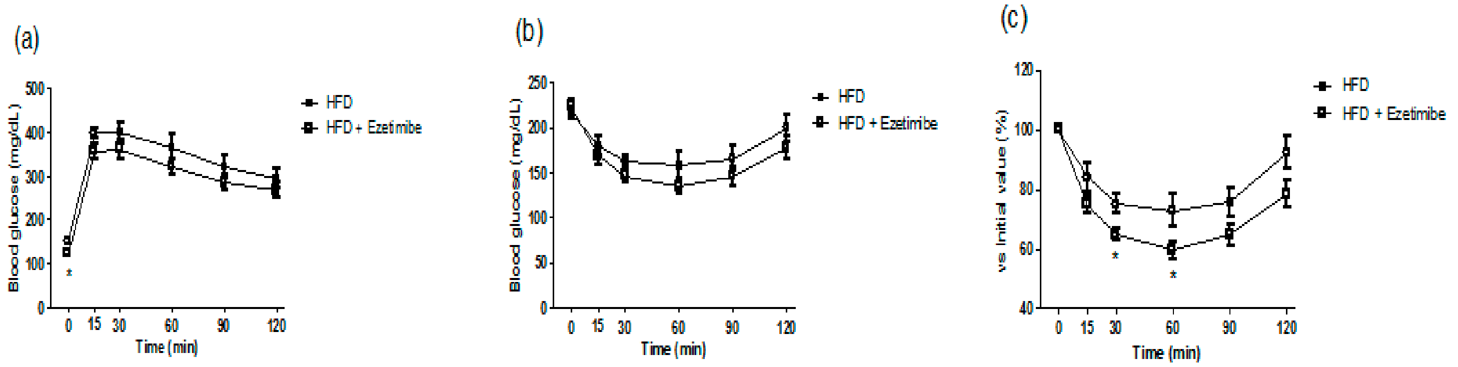

(a) Oral glucose tolerance test, (b) insulin tolerance test, (c) insulin tolerance test (vs initial value %). Error bars represent standard error of mean.
*$p < 0.05$ versus corresponding HFD value.
HFD, high fat diet

Method : In vivo (mouse model, pilot study) study

Thirty-nine male four-week-old C57BL/6J mice were fed a standard diet (5% wt/wt fat) to adapt to the environment. At 5 weeks of age, mice were randomly assigned to receive HFD with ezetimibe (45 Kcal% diet containing 0.004% w/w ezetimibe) in the ezetimibe group (n=21) or a HFD (45 Kcal%) in the control group (n=18). The total observation period was 19 weeks. Daily weight, dietary intake, activity patterns, and health status were monitored throughout the experiment. Fasting blood glucose levels were measured at baseline and after 19 weeks of drug administration. Oral glucose tolerance test was performed after 17 weeks of drug administration, when diabetes was developed. After fasting for 12 hours, 2 g/kg of glucose was administered orally, and blood glucose was measured from caudal venous blood by portable blood glucose meter at 0, 15, 30, 60, 90, and 120 minutes post glucose administration. Insulin tolerance test was performed one week after OGTT (after 18 weeks of drug administration). After fasting for 4 hours, 1 U/kg of insulin (Sigma-Aldrich, Cat. No. 9177C) was administered intraperitoneally, and blood glucose was measured from caudal venous blood by portable blood glucose meter at 0, 15, 30, 60, 90, and 120 minutes post insulin administration.

Supplementary figure 2. The expression of hepatic NPC1L1 on mice and rat liver

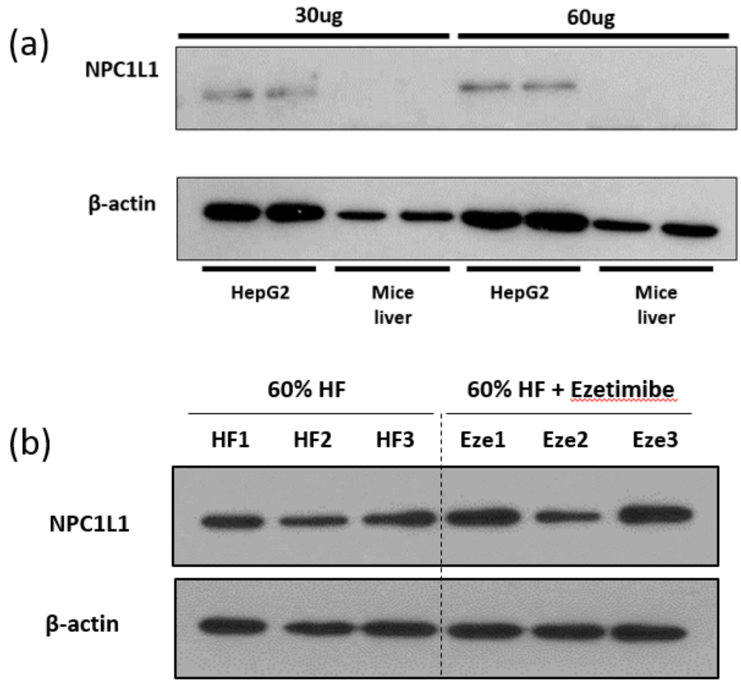

(a) Mice liver, HepG2 cells, and (b) rat liver (high fat diet with / without ezetimibe) were separated, homogenized with lysis buffer, and gel electrophoresis was performed. Western blotting was performed using an anti-NPC1L1 antibody against the antigen and actin (control) to determine the expression of NPC1L1 in mice and rat liver.

Supplementary figure 3. Effects of ezetimibe on the phosphorylation levels of Akt and the markers of gluconeogenesis in the HepG2 cells

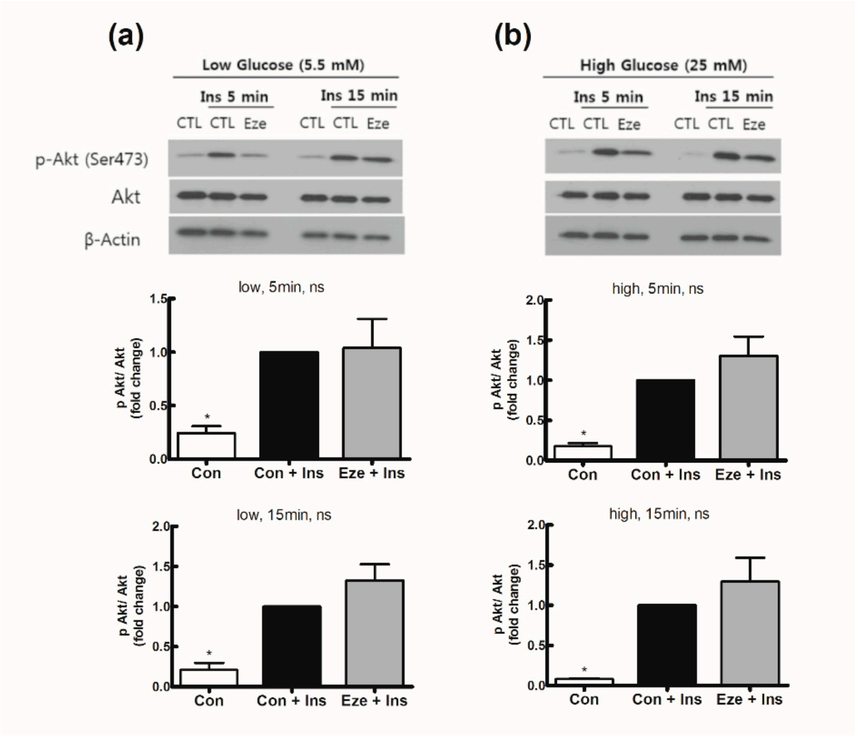

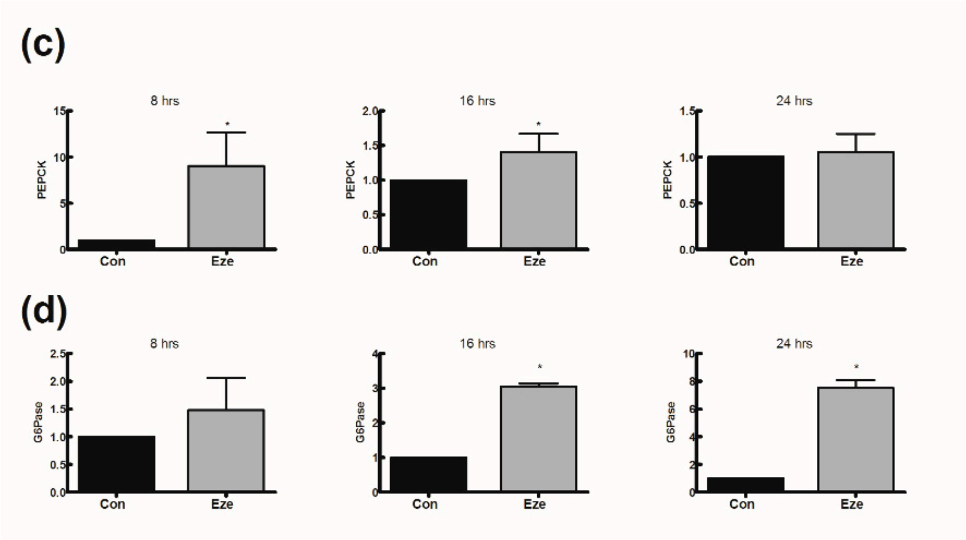

Representative western blots and plots of the densitometric analysis of the p-Akt/Akt ratio in the ezetimibe-treated cells, relative to that in the insulin-stimulated control cells (mean ± standard error (SE), n=3) at a (a)) high glucose concentration (25 mM). Plots of the densitometric analysis of the mRNA levels of (b) PEPCK and (c) G6pase mRNA relative to those of the control cells (mean ± SE, n=3). Compared using the Wilcoxon-Mann-Whitney U test; *$p < 0.05$

Supplementary figure 4. Effects of ezetimibe on markers of gluconeogenesis in HepG2 cells, evaluated by western blot

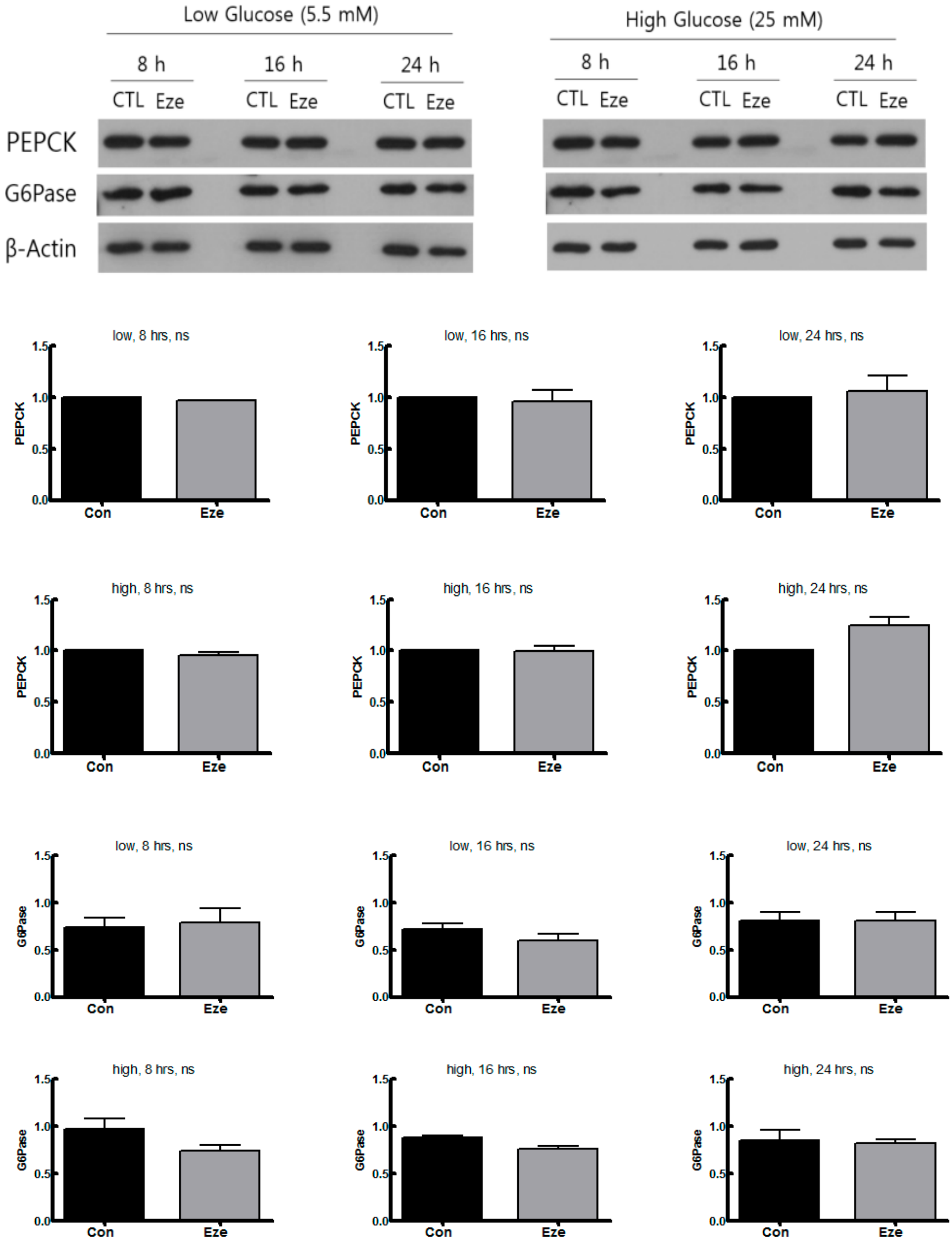

Above: Representative western blots. Below: plots of densitometric analysis of PEPCK and G6Pase in relation to insulin-stimulated control cells (means ±SE, n=3). Error bars represent standard error of mean. Compared by the Wilcoxon-Mann-Whitney U test. * $p < 0.05$

Supplementary Figure 5. Effects of ezetimibe on glucose outflow in HepG2 cells

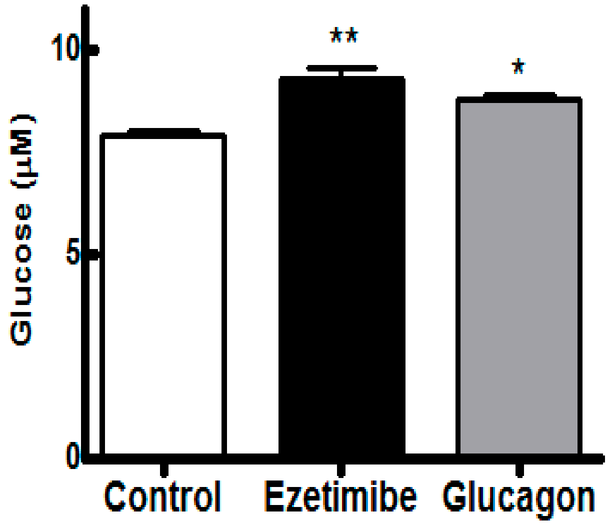

Plots of glucose outflow in HepG2 cells (means ±SE, n=12). Error bars represent standard error of mean. Compared by the ANOVA test. * $p < 0.05$, ** $p < 0.01$

Supplementary figure 6. Changes of homeostatic model assessment of insulin resistance in patients of each group

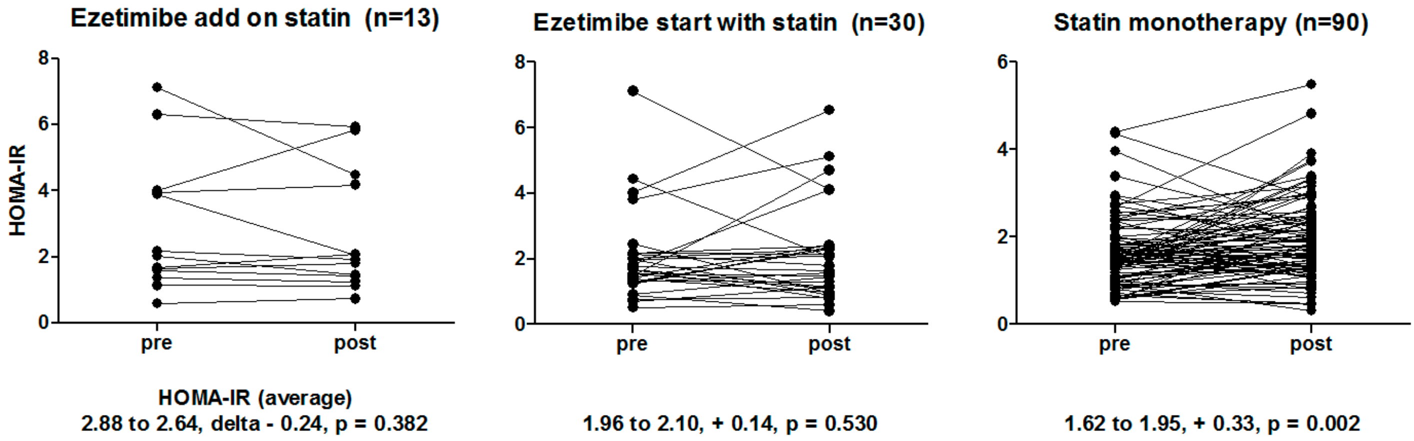

Supplement: Supplementary file 1 [file biomedicines-08-00512-s001.pdf]
